# Revisiting the continuum of resistance model in the digital age: a comparison of early and delayed respondents to the Norwegian counties public health survey

**DOI:** 10.1186/s12889-021-10764-2

**Published:** 2021-04-15

**Authors:** Benjamin Clarsen, Jens Christoffer Skogen, Thomas Sevenius Nilsen, Leif Edvard Aarø

**Affiliations:** 1 Department of Health Promotion, Norwegian Institute of Public Health, Zander Kaaes Gate, 5017 Bergen, Norway; 2 Oslo Sports Trauma Research Centre, Norwegian School of Sport Sciences, Oslo, Norway; 3 Department of Public Health, Faculty of Health Sciences, University of Stavanger, Stavanger, Norway; 4 Alcohol & Drug Research Western Norway, Stavanger University Hospital, Stavanger, Norway; 5 Department of Health Studies, Norwegian Institute of Public Health, Oslo, Norway

**Keywords:** Epidemiologic methods, Selection bias, Surveys and questionnaires

## Abstract

**Background:**

The continuum of resistance model’s premise is that delayed respondents to a survey are more similar to non-respondents than early respondents are. For decades, survey researchers have applied this model in attempts to evaluate and adjust for non-response bias. Despite a recent resurgence in the model’s popularity, its value has only been assessed in one large online population health survey.

**Methods:**

Respondents to the Norwegian Counties Public Health Survey in Hordaland, Norway, were divided into three groups: those who responded within 7 days of the initial email/SMS invitation (wave 1, *n* = 6950); those who responded after 8 to 14 days and 1 reminder (wave 2, *n* = 4950); and those who responded after 15 or more days and 2 reminders (wave 3, *n* = 4045). Logistic regression analyses were used to compare respondents’ age, sex and educational level between waves, as well as the prevalence of poor general health, life dissatisfaction, mental distress, chronic health problems, weekly alcohol consumption, monthly binge drinking, daily smoking, physical activity, low social support and receipt of a disability pension.

**Results:**

The overall response to the survey was 41.5%. Respondents in wave 1 were more likely to be older, female and more highly educated than those in waves 2 and 3. However, there were no substantial differences between waves for any health outcomes, with a maximal prevalence difference of 2.6% for weekly alcohol consumption (wave 1: 21.3%, wave 3: 18.7%).

**Conclusions:**

There appeared to be a mild continuum of resistance for demographic variables. However, this was not reflected in health and related outcomes, which were uniformly similar across waves. The continuum of resistance model is unlikely to be useful to adjust for nonresponse bias in large online surveys of population health.

**Supplementary Information:**

The online version contains supplementary material available at 10.1186/s12889-021-10764-2.

## Background

Differences are likely between people that respond to public health surveys and those that do not. Among non-respondents, there is commonly a disproportionate number of young [1–8], male [2–10], and unmarried people [1, 2, 5, 8, 11–14], as well as those with lower education [1, 2, 5–8, 10, 12–15], and lower socioeconomic status [5, 6, 8, 11, 13, 16]. Non-respondents are also more likely to be smokers [1, 4, 10, 14, 17–19], and to have different patterns of alcohol consumption [10, 16, 20–22], poorer physical and/or mental health [5, 7, 9, 10, 23], and higher rates of mortality and morbidity [20, 24–29]. If researchers fail to account for nonresponse bias, prevalence estimates (in particular) and analyses of associations between variables will likely be incorrect [9].

Many survey investigators hope to reduce the risk of nonresponse bias by achieving a high response rate, and often send multiple reminders to non-respondents, encouraging them to participate. Nevertheless, few large-scale public health surveys achieve a response rate adequate to reduce the likelihood of substantial nonresponse bias, which by some estimates is between 70 and 90% [30]. As participation rates in epidemiologic studies have been declining over time [2, 31], and because nonresponse bias can exist even when response rates are high [32], it is increasingly important for researchers to identify and account for nonresponse bias when summarising and analysing data. Obviously, this is a major challenge because information on non-respondents is often unavailable, particularly for the outcomes of interest.

Researchers have sought methods to identify nonresponse bias for decades. In 1939, Pace proposed that the existence and direction of nonresponse bias in a given survey could be detected by comparing the responses of people who respond quickly (early respondents) to those who only respond after repeated contact attempts (delayed respondents) [33]. This approach, often referred to as the “continuum of resistance” model [34], is based on the presumption that people who are slow or reluctant (i.e. resistant) to complete a questionnaire are more similar to non-respondents than early respondents are.

The continuum of resistance model has resurfaced periodically in the literature since its proposal, despite having performed inconsistently under empirical testing. Some early studies supported the existence of a continuum of resistance for outcomes of interest [35, 36]; however, others have found that early and delayed respondents do not differ at all [37, 38], that associations between delayed respondents and non-respondents are weak [34], or that a continuum of resistance exists for demographic variables but not for outcomes of interest [39]. Recently, the model has been applied in a number of surveys of health-related behaviours [3, 13, 40–43], and in one national public health survey [44]. In several studies on drug and alcohol use, researchers identified significant and consistent differences between early and late respondents for both demographic variables and outcomes of interests. Subsequently, delayed respondents’ data was used to adjust prevalence estimates to account for nonresponse bias [3, 13, 40, 42, 43].

Given the apparent value of the continuum of resistance model in these recent studies, and because the model has only been applied once in a large online survey of population health [44], we compared early and delayed respondents of the internet-based Norwegian Counties Public Health Survey. We hypothesized that, compared to early respondents, late respondents would be more likely to be male, young and less educated, and that they would have a higher prevalence of poor health outcomes and behaviours associated with poor health.

## Methods

### Study design and setting

The Norwegian Counties Public Health Survey is an online cross-sectional study of self-reported health, health-related behaviours, quality of life, and local health-related factors in the Norwegian general population. The survey was launched by the Norwegian Institute of Public Health in 2015 and is currently ongoing, covering each of Norway’s 11 counties every 4 years. We performed this investigation using data collected in the county of Hordaland between the 10th of April and the 13th of June 2018. The survey was approved by the Norwegian Data Protection Authority. This study is a secondary analysis of previously collected data, and according to the Norwegian Health Research Act, additional ethical approval was not required.

### Participants

A random sample of 38,458 Hordaland County residents was selected from the National Population Register and invited to participate in this survey. To be eligible for the sampling frame, residents needed to be over 18 years of age and have their mobile telephone number and email address registered with the Norwegian Agency for Public Management and eGovernment. This contact register includes approximately 80% of Norwegian residents aged between 18 and 65 years and 50% of those aged over 75 years [45]. The sample size was determined to allow for subgroup analysis on a municipal level (minimum of 400 participants per subgroup), with oversampling of the smallest municipalities and an expected overall response of 30 to 40%.

The questionnaire, which could be completed using a PC, tablet or smartphone, was distributed to the sample by email and short message service (SMS) using a secure platform [46]. Non-respondents received email and SMS reminders with a link to the questionnaire on the 7th and 15th day after the initial distribution, and the survey remained open for 5 weeks and 3 days.

### Variables

- **Response to questionnaire invitation:** All members of the invited sample were categorised as survey *respondents* or *non-respondents*. Respondents were further categorised into one of three groups based on when they completed the questionnaire: (1) *Wave 1* - completed the questionnaire prior to the first reminder; (2) *Wave 2* - completed the questionnaire between the first and second reminders; (3) *Wave 3* - completed the questionnaire after the second reminder.
- **Gender:** Male or female, as recorded in the national population register.
- **Age:** Categorised as *18–29*, *30–39*, *40–49*, *50–59*, *60–69* and *70 or older*
- **Education**: Respondents were categorised according to their on highest-attained level of education: *junior high school* (up to and including 10th grade), *senior high school* (up to and including 13th grade), *university or university college for less than 4 years*, and *university or university college for 4 or more years*
- **Poor general health:** Respondents were categorised as having poor general health if they reported having bad or very bad general health on a 5-point scale including *very good, good, neither good nor bad, bad* and *very bad*.
- **Dissatisfied with life:** Respondents were categorised as being dissatisfied with life if they reported being quite dissatisfied or very dissatisfied on a 5-point scale with the alternatives *very satisfied, quite satisfied, neither satisfied nor dissatisfied, quite dissatisfied*, and *very dissatisfied*.
- **Mental distress:** Based on the five items’ version of the Hopkins Symptom Checklist (HSCL-5), with four response options ranging from *not at all* (1 point) to *extremely* (4 points). We classified respondents with a mean item score greater than 2 as having high levels of mental distress [47].
- **Chronic health problems:** Respondents who reported having a chronic health problem or disability that has lasted at least 6 months (including seasonal and intermittent problems).
- **Alcohol consumption:** Based on the consumption questions of the Alcohol Use Disorders Identification Test (AUDIT) [48]. Respondents were categorised into those who did and did not drink alcohol more than once a week, and those who did and did not consume six or more standard drinks (10 g ethanol) in a single session more than once a month (monthly binge drinkers).
- **Daily smoking:** Respondents who reported that they smoked tobacco on a daily basis
- **Physical activity:** Based on the International Physical Activity Questionnaire (IPAQ). Respondents were classified as being physically active if they performed moderate or vigorous physical activity daily or walked for at least 30 min every day.
- **Low social support:** Respondents who reported that they experienced low social support using a previously described categorisation of the Oslo-3 Social Support Scale (OSS-3) [49].
- **Receiving disability pension:** Respondents who reported that they currently receive a disability pension.

### Statistical analyses

First, to identify how questionnaire response varied by age and gender, we calculated response rates separately for each response wave by age category and gender. We then assessed age and gender differences between respondents and non-respondents by comparing the age category and gender distribution of the invited sample with the distributions within each response wave and the distribution among non-respondents (made possible by information from the national population register). To assess educational differences between waves, we were limited to comparing the proportion of respondents reporting each level of education within each response wave (as information on non-respondents’ educational level was not available). Finally, to assess for a potential continuum of resistance in our data, we assessed associations between respondents’ wave (independent variable) and general health, life satisfaction, mental distress, chronic health problems, alcohol consumption, smoking, physical activity, social support, and receiving a disability pension (dependent variables). These were performed both as univariate analyses and as multivariable analyses which included age and sex as covariates.

We performed binomial logistical regression in analyses involving dichotomous dependent variables, and multinomial regression when dependent variables had more than two outcome categories. Weighting was used to correct for oversampling of small municipalities. All analyses were conducted in R [50]. We used the packages nnet to fit multinomial log-linear models [51], margins and effects to generate marginal effects [52–54], and ggplot2 to produce figs [55]. A 95% confidence interval (CI) was calculated for all estimates, and we used a significance threshold of .05.

## Results

**Fig. 1:**
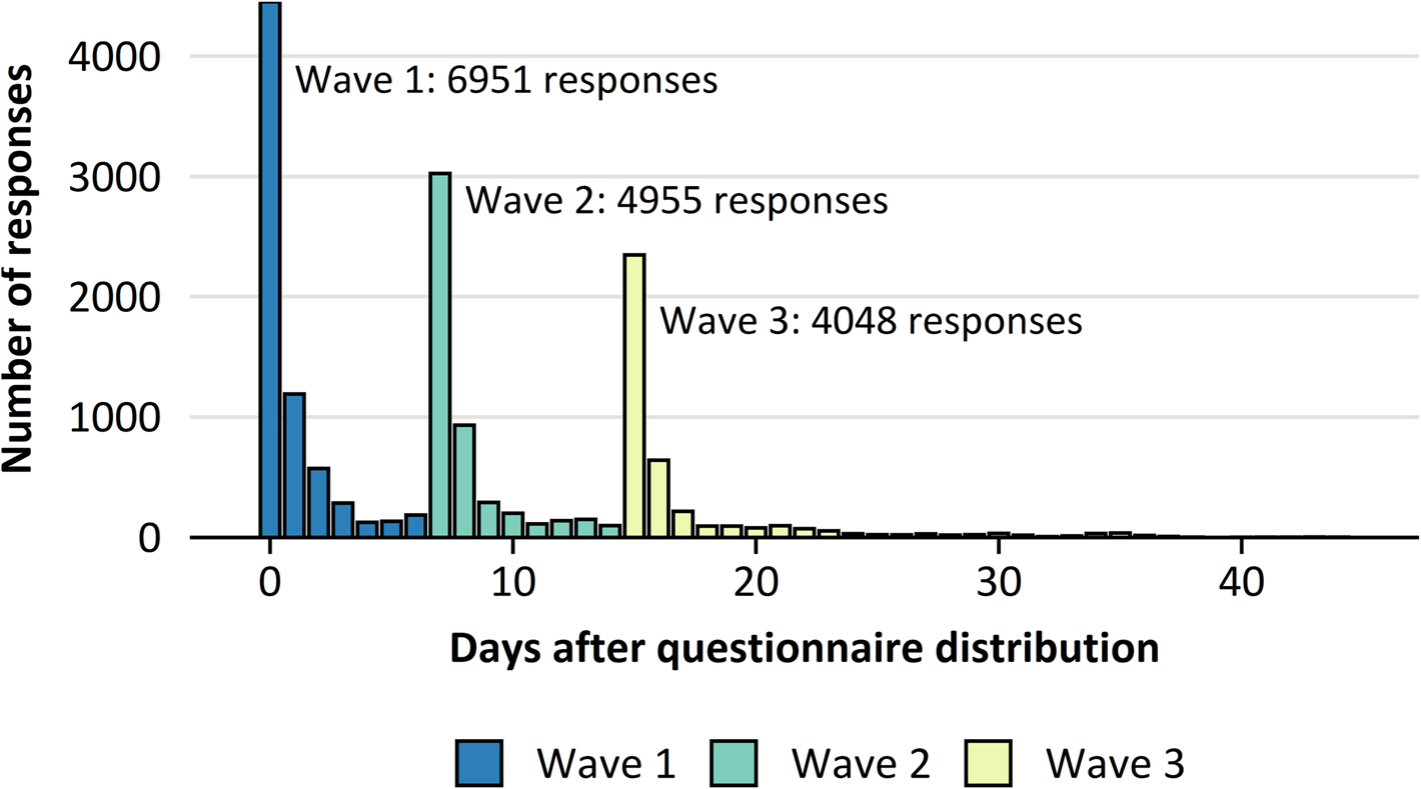
Number of questionnaire responses on each the day of the survey and in each response waves: Wave 1 (0–7 days from the initial questionnaire distribution), Wave 2 (8–14 days) and Wave 3 (15–45 days). Reminders to complete the questionnaire were distributed on days 8 and 15. Had the survey not used a second reminder, it is likely that many respondents of the third wave would have remained non-respondents

**Fig. 2:**
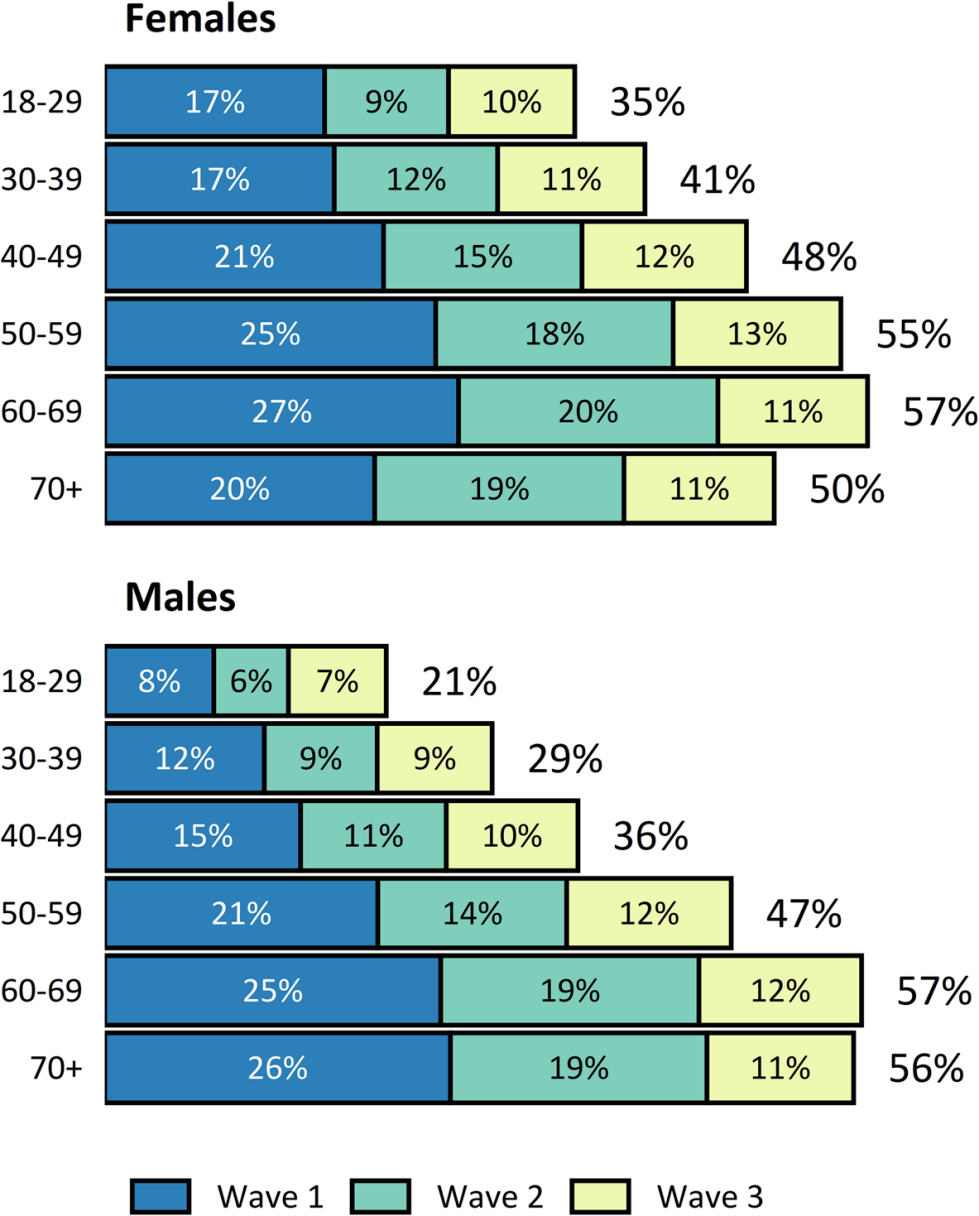
Questionnaire response rate by age group and gender. The overall response rate is shown to the right of each bar, and the response rate for each wave is shown within each segment of the bars

The overall response rate to the questionnaire was 41.5%, with 44% of responses received in wave 1, 31% in wave 2, and 25% in wave 3 (Fig. 1). The response rate was substantially higher among females (46%) than among males (37%), and it was higher in older age groups (Fig. 2).

**Fig. 3:**
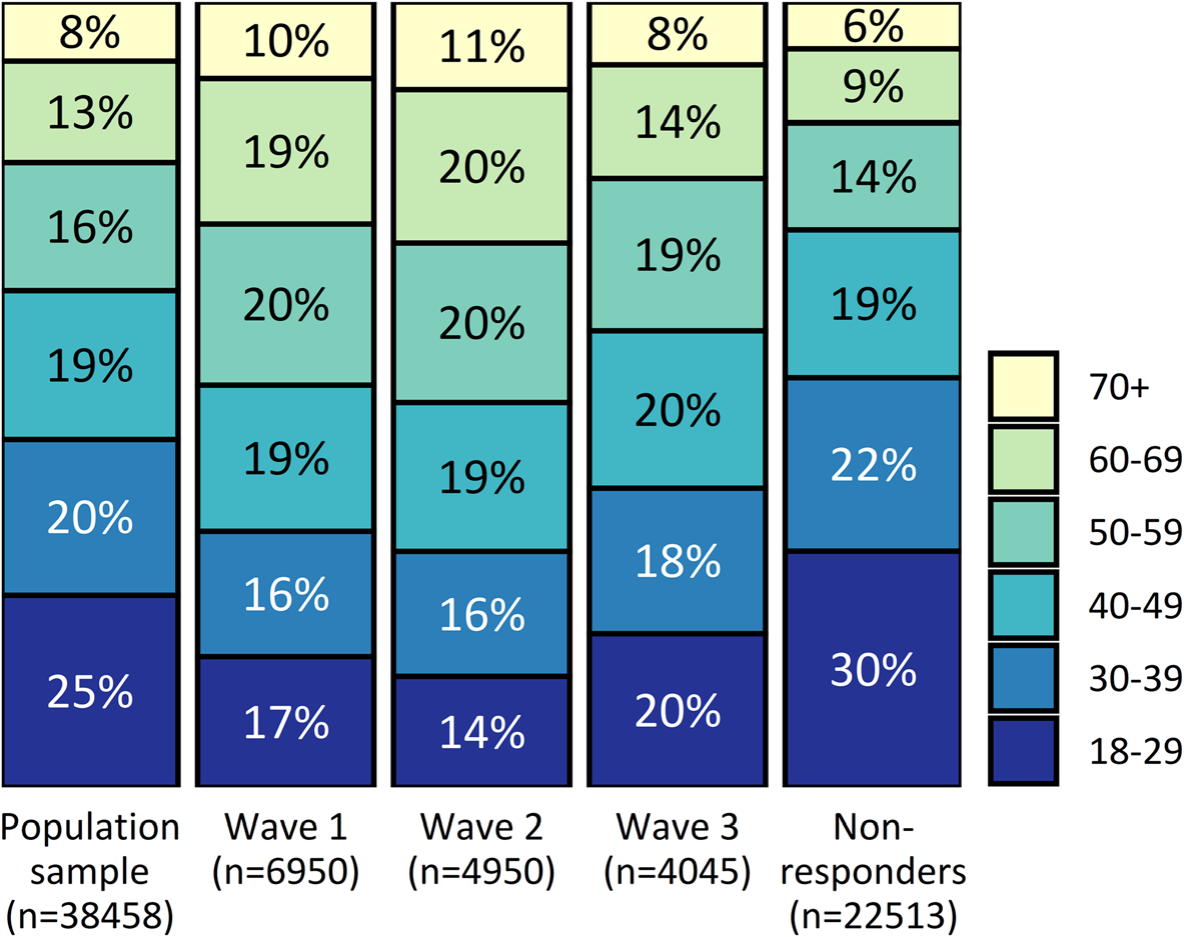
Age group proportions within the invited sample, each response wave, and non-respondents

**Fig. 4:**
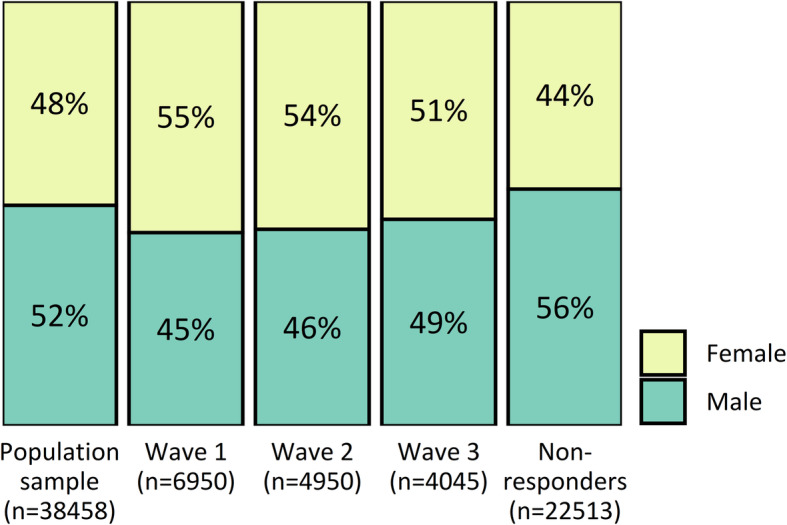
Proportion of males and females within the invited sample, each response wave, and non-respondents

The age distribution of the invited sample, each response wave and non-respondents is shown in Fig. 3. Younger people were under-represented and older people were over-represented among respondents, particularly in waves 1 and 2. Similarly, males were under-represented among respondents, particularly in waves 1 and 2 (Fig. 4).

**Fig. 5:**
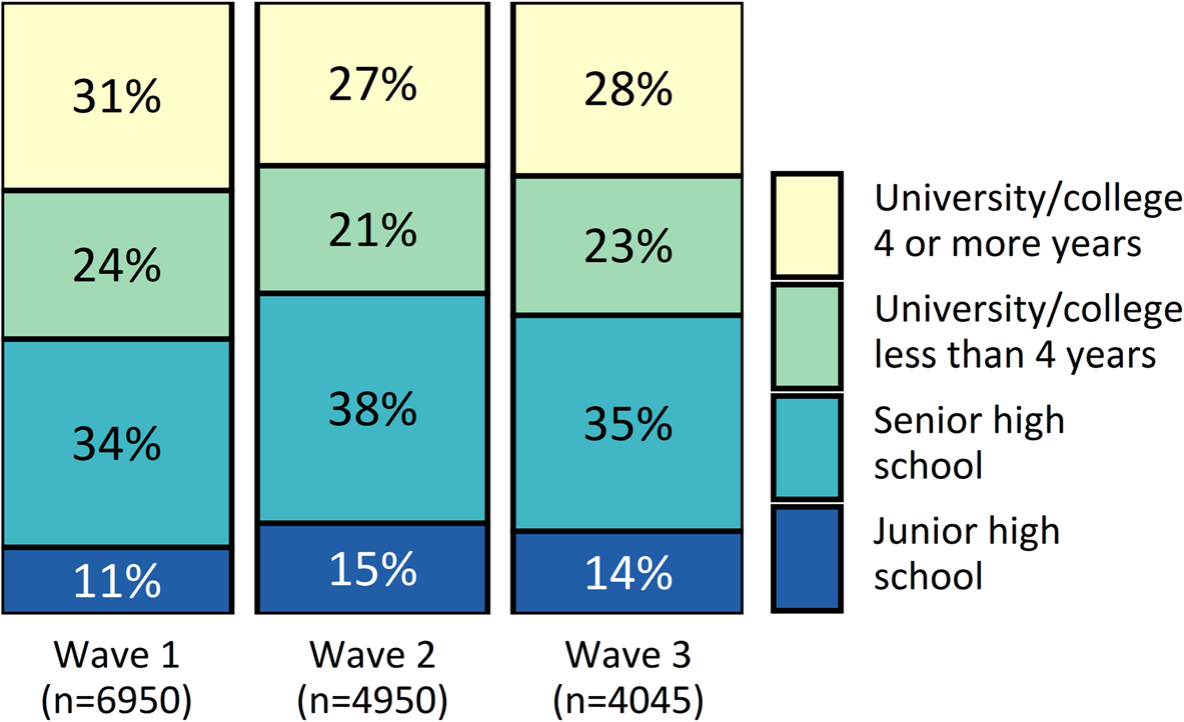
Distribution of educational level in each response wave

There were small differences in the distribution of respondents’ educational level between each wave, with wave 1 respondents having a relatively higher level than wave 2 and 3 respondents (Fig. 5).

### Health outcomes

**Fig. 6:**
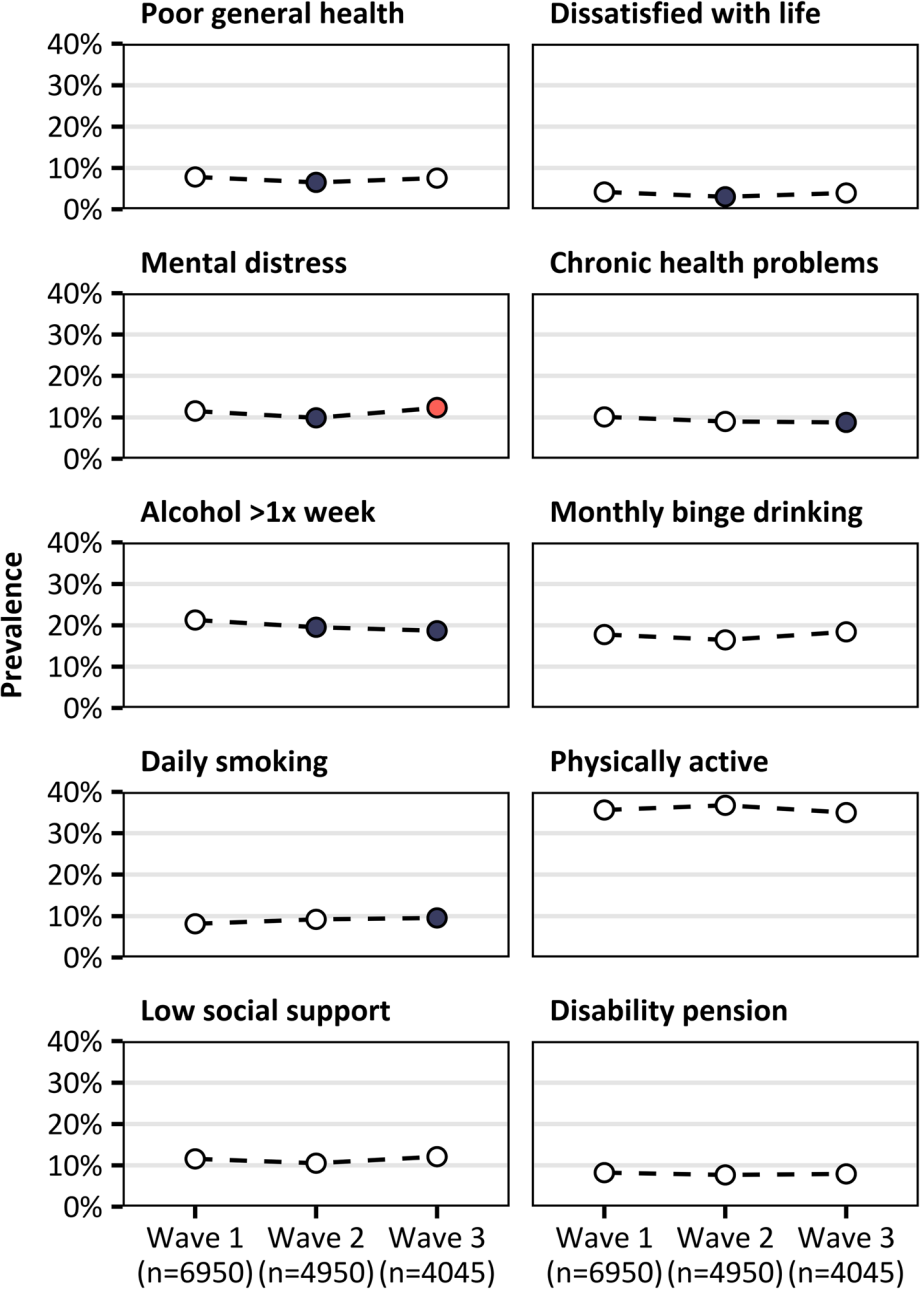
Prevalence of various health and health-related outcomes among questionnaire respondents in waves 1, 2 and 3. Red dots indicate significant differences from wave 1 (*p* < 0.05) and the black dot indicates a significant difference between wave 2 and wave 3 (*p <* 0.05)

Table 1 and Fig. 6 show the proportion of respondents in each wave that reported poor general health, life dissatisfaction, mental distress, chronic health problems, drinking alcohol more than once per week, monthly binge drinking, daily smoking, physical activity, low social support, and receiving a disability pension. Table 2 shows the results of pairwise comparisons of each wave using logistic regression analyses. Wave 2 respondents had, in comparison to wave 1, a lower prevalence of poor general health, life dissatisfaction, mental distress, and weekly alcohol consumption. Wave 3 respondents had, in comparison to wave 1, a higher prevalence of mental distress and daily smoking, and a lower prevalence of chronic health problems and weekly alcohol consumption. Additionally, wave 3 respondents had a higher prevalence of mental distress than wave 2 respondents did. All differences between groups were small, with the maximum absolute prevalence difference between the highest and lowest waves being 2.6% (weekly alcohol consumption, Table 1).

**Table 1:** Prevalence (%, [95% CI]) of health and health-related outcomes among respondents in waves 1, 2 and 3

	Wave 1	Wave 2	Wave 3
Poor general health	7.8 [7.1, 8.5]	6.5 [5.7, 7.3]*	7.5 [6.6, 8.5]
Dissatisfied with life	4.2 [3.7, 4.7]	3.0 [2.5, 3.6]*	3.9 [3.3, 4.6]
Mental distress	11.5 [10.7, 12.4]	9.9 [9.0, 10.9]*	12.3 [11.2, 13.5]^#^
Chronic health problems	10.1 [9.3, 10.9]	9.0 [8.1, 9.9]	8.8 [7.8, 9.8]*
Alcohol >1x week	21.3 [20.2, 22.4]	19.5 [18.3, 20.8]*	18.7 [17.3, 20.0]*
Monthly binge drinking	17.8 [16.7, 18.8]	16.5 [15.3, 17.7]	18.4 [17.0, 19.8]
Daily smoking	8.2 [7.4, 8.9]	9.2 [8.3, 10.1]	9.6 [8.6, 10.6]*
Physically active	35.6 [34.3, 37.0]	36.8 [35.1, 38.4]	35.0 [33.2, 36.8]
Low social support	11.6 [10.7, 12.4]	10.5 [9.6, 11.5]	12.1 [11.0, 13.2]
Disability pension	8.2 [7.5, 9.0]	7.7 [6.9, 8.5]	7.9 [7.0, 8.9]

* Sig. different to wave 1 (*p <* 0.05)

^#^ Sig. difference between wave 2 and wave 3 (*p* < 0.05)

**Table 2:** Results of logistic regression models (odds ratios [95% confidence interval])

	Wave 1 (ref.)	Wave 2	Wave 3
Poor general health	1.00	0.82 [0.70, 0.96]*	0.96 [0.82, 1.13]
Dissatisfied with life	1.00	0.72 [0.57, 0.90]*	0.94 [0.75, 1.17]
Mental distress	1.00	0.84 [0.74, 0.97]*	1.08 [0.94, 1.24]
Chronic health problems	1.00	0.88 [0.77, 1.01]	0.86 [0.74, 1.00]*
Alcohol >1x week	1.00	0.90 [0.81, 0.99]*	0.85 [0.76, 0.95]*
Monthly binge drinking	1.00	0.91 [0.82, 1.02]	1.05 [0.93, 1.17]
Daily smoking	1.00	1.14 [0.99, 1.32]	1.19 [1.02, 1.39]*
Physically active	1.00	1.05 [0.96, 1.15]	0.97 [0.88, 1.07]
Low social support	1.00	0.90 [0.79, 1.03]	1.05 [0.92, 1.20]
Disability pension	1.00	0.93 [0.80, 1.08]	0.96 [0.82, 1.13]

*p < 0.05

Our findings were similar when age and sex were included as covariates in these analyses (see supplementary material).

## Discussion

Survey researchers have retained interest in the continuum of resistance model for 80 years, despite conflicting evidence of its validity. Today, with response rates generally declining [2, 31], finding effective ways to assess nonresponse bias is as important as ever.

We applied the continuum of resistance model to a large online public health survey, comparing respondents who completed the questionnaire within the first 7 days (wave 1), those who completed it after 8 to 15 days and one reminder (wave 2) and those who completed it after 16 or more days and two reminders (wave 3). For demographic variables, we identified differences between waves that were consistent with previous literature [1–10, 12–15]. However, any differences in health outcomes and behaviours were small between waves and were unlikely to be useful in identifying nonresponse bias. Overall, females and older people were more likely to respond to the questionnaire than males and younger people were. This was most pronounced among wave 1 and 2 respondents, whereas wave 3 more closely resembled the invited sample, containing a higher proportion of males and younger people. However, it is important to note that for sex and age, the composition of wave 3 more closely resembled waves 1 and 2 than it did the non-respondents.

For education, our findings were similar. There was a slight trend towards wave 1 respondents being more highly educated than those in wave 2 and 3. However, the difference between respondents and non-respondents is likely to be much larger than the small differences between response waves. Although we lacked direct information on the education level of non-respondents, data from Statistics Norway show that 35% of Hordaland county residents have tertiary education, and that 24% have only completed junior high school [56]. These proportions differ markedly from our results (52 and 13%, respectively), suggesting that non-respondents had far lower levels of education than respondents did.

Based on the continuum of resistance model, we expected that late respondents would display an overall pattern of poorer health across health outcomes. This has been found in a number of recent studies. For example, compared to early respondents, late respondents have been found to have a 21 to 68% higher prevalence of monthly binge drinking [3, 40, 42, 43], a 30% higher prevalence of current smokers [57], and a 50% higher prevalence of people who complete less than 30 min per day of physical activity [40]. We aligned our outcome definitions to facilitate comparisons with these studies, but did not find the same results. There was no difference between waves in the prevalence of monthly binge drinking or physical inactivity, and for current smoking, the difference in prevalence between waves 1 and 3 was only 1.4 percentage points. Our findings were similar for other health outcomes; in some cases, there were statistically significant but very small prevalence differences between waves, and in others there were none.

Our findings are supported by a recent comparison of early and late respondents to a national online health survey in the Netherlands [44]. In that study, only small differences in health-related outcomes were identified between response waves, despite substantial differences in socio-demographic variables between waves. Further, when analyses were adjusted for sociodemographic variables, the differences in health-related outcomes all but disappeared. In our results, there was little change when age and sex were adjusted for.

There are several potential explanations for why we did not find evidence to support a continuum of resistance in our data. Indeed, it is possible that the health status of respondents and non-respondents is very similar in our population. We believe this is unlikely, particularly considering the findings of Knudsen et al., who, in 2008, reported a substantially higher prevalence of mental and somatic health disorders among non-respondents to a health survey conducted in Hordaland county [9]. Our definition of late respondents differs from some recent studies demonstrating a continuum-of-resistance, which have used more reminders [43], longer follow-up periods [3, 12, 40–42], and/or alternative methods such as telephone calls to contact slow respondents [3, 41, 42]. To our knowledge, this is the only continuum-of-resistance study besides those of Kypri et al. [40, 41] and Klingwort [44] to collect data using purely digital means. It is possible that the barriers to questionnaire completion differ between postal, telephone and internet/smartphone surveys, and that the data collection method has consequences on any eventual continuum of resistance.

This study has several limitations that we were unable to account for, and that may have affected our findings. First, we had no information about the health status of non-respondents, but rather we assumed that there were differences based on previous research. Future studies linking survey data with other sources, such as national registers, are necessary to gain more information on the health status of non-respondents. Additionally, to be eligible for inclusion in the survey, people had to have their digital contact information registered with the Norwegian authorities. This introduces a selection bias that is particularly pronounced among older people [45]. It is therefore likely that the health status of the survey sample is more homogeneous than it is in the general population.

## Conclusion

We found that keeping the survey open for an extended period and using multiple reminders increased the overall proportion of male, younger and less-educated respondents. However, we were unable to identify meaningful differences in reported health and health determinants between early and late survey respondents. Assuming there are true differences in the health status of respondents and non-respondents, the results of delayed respondents provided little help in estimating the direction or magnitude of non-response bias.

## Supplementary Information

**Additional file 1.**

## Data Availability

Public access to the database is closed. However, data may be available to researchers with study questions that fall within the general aims of Norwegian Counties Public Health Survey. Applicants and projects must fulfill requirements in Norwegian regulations and laws concerning research and protection of personal information (GDPR). Enquiries can be sent to fylkeshelseundersokelser@fhi.no.

## Abbreviations

SMS: Short Message Service
HSCL-5: Hopkins Symptom Checklist - 5
AUDIT: Alcohol Use Disorders Identification Test
IPAQ: International Physical Activity Questionnaire
OSS-3: Oslo-3 Social Support Scale
