# Supplementary material for: Revisiting the continuum of resistance model in the digital age: a comparison of early and delayed respondents to the Norwegian counties public health survey
Source: BMC Public Health. 2021 Apr 15;21:730. doi: 10.1186/s12889-021-10764-2 (PMC8048233; doi:10.1186/s12889-021-10764-2)
Supplement: Supplementary file 1 — Additional file 1. [file 12889_2021_10764_MOESM1_ESM.docx]

Revisiting the continuum of resistance theory in the digital age: A comparison of early and delayed respondents to the Norwegian Counties Public Health Survey

# Supplementary material

## Results of multivariable logistic regression analyses

| **Table S1.** Prevalence (%, [95% CI]) of health and health-related outcomes among respondents in waves 1, 2 and 3, adjusted for age and sex | | | |
| --- | --- | --- | --- |
|  | Wave 1 | Wave 2 | Wave 3 |
| Poor general health | 8.2 [7.5, 9.0] | 6.9 [6.1, 7.7]* | 8.1 [7.1, 9.1] |
| Dissatisfied with life | 3.1 [2.6, 3.6] | 2.3 [1.9, 2.7]* | 2.8 [2.3, 3.3] |
| Mental distress | 10.8 [9.9, 11.7] | 9.6 [8.6, 10.5]* | 10.9 [9.8, 12.0]^#^ |
| Chronic health problems | 11.3 [10.4, 12.2] | 10.1 [9.1, 11.1] | 10.1 [9.0, 11.2] |
| Alcohol >1x week | 17.3 [16.2, 18.3] | 15.2 [14.1, 16.3]* | 15.6 [14.4, 16.8]* |
| Monthly binge drinking | 10.2 [9.4, 11.0] | 9.4 [8.6, 10.3] | 9.6 [8.7, 10.6] |
| Daily smoking | 8.0 [7.2, 8.7] | 8.9 [8.0, 9.8] | 9.7 [8.6, 10.7]* |
| Physically active | 37.2 [35.7, 38.6] | 38.7 [37.0, 40.4] | 36.5 [34.7, 38.4] |
| Low social support | 9.9 [9.1, 10.7] | 9.2 [8.1, 10.1] | 10.0 [9.0, 11.0] |
| Disability pension | 9.8 [8.9, 10.7] | 9.2 [8.2, 10.1] | 9.9 [8.9, 11.0] |
| * Sig. different to wave 1 (p<0.05)  ^#^ Sig. difference between wave 2 and wave 3 (p<0.05) | | | |

| **Table S2.** Results of multivariable logistic regression models, adjusted for age and sex  (odds ratios [95% confidence interval]) | | | |
| --- | --- | --- | --- |
|  | Wave 1 (ref.) | Wave 2 | Wave 3 |
| Poor general health | 1.00 | 0.83 [0.71, 0.96]* | 0.98 [0.85, 1.14] |
| Dissatisfied with life | 1.00 | 0.74 [0.60, 0.91]* | 0.89 [0.73, 1.08] |
| Mental distress | 1.00 | 0.87 [0.77, 0.99]* | 1.01 [0.89, 1.14] |
| Chronic health problems | 1.00 | 0.89 [0.78, 1.01] | 0.89 [0.77, 1.02] |
| Alcohol >1x week | 1.00 | 0.86 [0.78, 0.94]* | 0.88 [0.80, 0.98]* |
| Monthly binge drinking | 1.00 | 0.92 [0.83, 1.02] | 0.94 [0.84, 1.05] |
| Daily smoking | 1.00 | 1.13 [0.99, 1.29] | 1.24 [1.08, 1.42]* |
| Physically active | 1.00 | 1.07 [0.98, 1.16] | 0.97 [0.89, 1.06] |
| Low social support | 1.00 | 0.92 [0.82, 1.04] | 1.00 [0.89, 1.13] |
| Disability pension | 1.00 | 0.93 [0.81, 1.06] | 1.01 [0.87, 1.17] |
| *p<0.05 | | | |
